# Long COVID Patients with Orthostatic Intolerance Have Reduced Heart Rate Variability and Preserved Physiological Response to Active Standing

**DOI:** 10.3390/biology15010001

**Published:** 2025-12-19

**Authors:** J. Antonio González-Hermosillo González, Claudia Lerma, Dulce Andrea Celestino Montelongo, María del Carmen Alba Lorenzo, Emiliano Salas Santos, Atziri Gun Cuninghame Ballesteros, Esteban Jorge-Galarza, María del Rocío Martínez-Alvarado

**Affiliations:** 1 Department of Cardiovascular Dysautonomia, Instituto Nacional de Cardiología Ignacio Chávez, Mexico City 14080, Mexico; sincope39@yahoo.com.mx (J.A.G.-H.G.); andreacelestmon@gmail.com (D.A.C.M.); malbalorenzo@gmail.com (M.d.C.A.L.); salas_emiliano@yahoo.com (E.S.S.); atziri.saoirse@gmail.com (A.G.C.B.); 2 Laboratory of Cardiovascular Dynamics, Department of Molecular Biology, Instituto Nacional de Cardiología Ignacio Chávez, Mexico City 14080, Mexico; dr.claudialerma@gmail.com; 3 Department of Outpatients Care, Instituto Nacional de Cardiología Ignacio Chávez, Mexico City 14080, Mexico; esjoga@yahoo.com.mx

**Keywords:** long COVID, heart rate variability, autonomic nervous system

## Abstract

The aim of this study was to assess the heart rate variability (HRV) at rest and during active orthostatic challenge in long COVID patients with orthostatic intolerance symptoms (dizziness, pre-syncope, and syncope). We performed a cross-sectional, observational, comparative study of 60 subjects of both sexes, aged 18 to 60 years (31 met the criteria of long COVID, 15 were infected individuals without symptoms, and 14 who had neither infection nor symptoms formed the age-matched control group). HRV was obtained from continuous electrocardiograms in a supine position and active standing with spontaneous breathing. The time from SARS-CoV-2 infection to testing in the COVID-19 group was 573 ± 289 days. The resting (supine position) values of SDNN, RMSSD, SD1, and SD2 were lower in long COVID patients than in control participants, while all other HRV indexes were similar between groups. In response to active standing, both groups had similar changes in all HRV indices. In conclusion, an active orthostatic test was not able to exhibit an autonomic dysregulation in these patients with long COVID, suggesting that cardiac autonomic modulation may have recovered due to the long time that elapsed after SARS-CoV-2 infection.

## Simple Summary

A substantial number of patients recovering from COVID-19 develop symptoms three months after infection, lasting at least two months and not explained by other diagnoses, a condition known as long COVID. They include symptoms of autonomic nervous system dysfunction: orthostatic intolerance (dizziness, fainting), fatigue, brain fog, headache, sleep problems, and palpitations. The present study assessed the autonomic nervous system modulation of the heart by performing an analysis of heart rate variability at rest and during standing in patients with long COVID and orthostatic intolerance compared to asymptomatic healthy people. The time from SARS-CoV-2 infection to testing in the COVID-19 group was 573 ± 289 days. Long COVID patients had lower heart variability at rest compared to healthy participants. In response to standing, both groups had similar physiological changes in heart rate variability. This indicates that patients with long COVID and orthostatic intolerance, despite having reduced heart rate variability at rest, do not show immediate autonomic dysregulation upon standing. These results warrant further studies to prove if the cardiac autonomic modulation may recover after the long elapsing time post-infection.

## 1. Introduction

Long COVID affects 10% to 60% of individuals after SARS-CoV-2 acute infection [1] and is characterized by persistent symptoms that typically emerge three months post-infection and last for at least two months without an alternative explanation [2]. These symptoms occur across all age groups and may follow even mild acute disease, though they are more common after severe COVID-19 [3]. Among the most frequent and disabling manifestations are exercise intolerance, dyspnea, fatigue, cognitive impairment, dizziness, and tachycardia, features that overlap substantially with cardiovascular autonomic dysfunction (CAD) [4,5].

Emerging evidence suggests that autonomic disturbances are common in long COVID, with reported prevalence ranging from 30% in formal autonomic testing to 75% using symptom-based instruments such as the COMPASS-31 questionnaire [6]. Orthostatic intolerance is particularly frequent [4,7], yet hemodynamic findings in active standing tests remain inconsistent across studies [7,8]. These disparities highlight the need for more precise characterization of autonomic responses in long COVID, particularly during physiologically relevant orthostatic stress.

Heart rate variability (HRV) offers a non-invasive method to assess autonomic modulation and has shown reduced parasympathetic activity in some long COVID cohorts [8,9]. However, HRV findings are heterogeneous, with several studies reporting preserved autonomic regulation in specific subgroups [10]. Importantly, previous investigations of HRV during orthostatic challenge in long COVID have relied exclusively on the head-up tilt test (HUTT) [11]. No studies in long COVID to date have evaluated HRV responses to active standing, despite its physiological validity and relevance for patients who report orthostatic symptoms [12,13,14]. Thus, the autonomic mechanisms underlying orthostatic intolerance in long COVID remain insufficiently characterized, particularly regarding dynamic HRV changes during active standing.

The present study addresses this gap by evaluating cardiac autonomic modulation, using HRV, during an active orthostatic challenge in patients with long COVID who report orthostatic symptoms, compared with healthy controls, and by relating these findings to concurrent hemodynamic abnormalities.

We hypothesize that, compared with healthy controls, individuals with long COVID and orthostatic intolerance may exhibit impaired parasympathetic withdrawal and/or exaggerated sympathetic activation during active standing, as reflected by distinctive HRV patterns and abnormal hemodynamic responses. However, given the heterogeneous and sometimes conflicting findings reported in the literature, and the long time that has elapsed after SARS-CoV-2 infection in many patients, a normal autonomic adjustment could not be excluded.

## 2. Materials and Methods

### 2.1. Study Design and Participants

This is a cross-sectional, observational, comparative study. A total of 63 subjects, of both sexes, aged 18 to 60 years, were recruited from April 2023 to October 2024. During this period in Mexico, the dominant SARS-CoV-2 variant was Omicron. Thirty-one patients with SARS-CoV-2 who experienced persistent symptoms such as fatigue, palpitations, dizziness upon standing, and syncope, which persisted for at least 3 months after recovering from acute COVID-19 infection, and met the criteria for long COVID, were included in this study [2].

**Figure 1:**
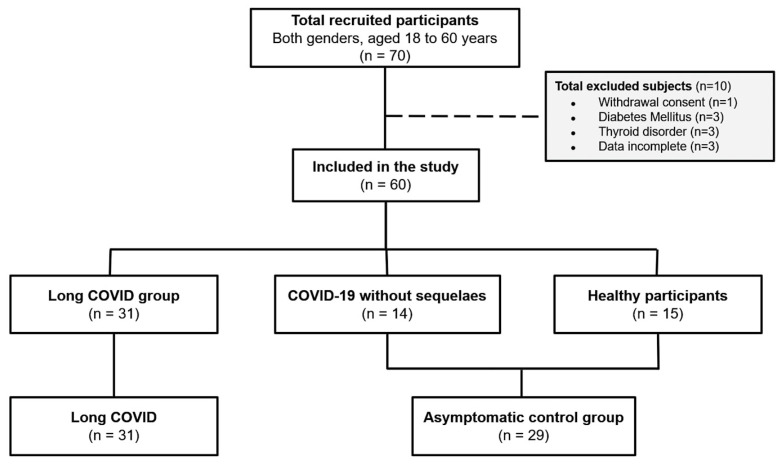
Flowchart of the study design.

A total of 15 patients with COVID-19 who did not report long COVID symptoms and 14 healthy individuals without COVID-19 (never had symptoms suggesting COVID-19 or tested positive with molecular tests) formed the age-matched control group (Figure 1). All participants who had COVID-19 (either with or without long COVID) reported mild-to-moderate infection. Four (12.9%) of the long COVID and one (3.4%) of the non-long COVID individuals were hospitalized. Only two of the long COVID patients and none of the non-long COVID patients required ventilation.

Participants with a diagnosis or suspicion of hypertension, a history of cancer or oncological treatments, endocrine disorders, chronic diseases such as diabetes mellitus, rheumatological, or neurological diseases, or those taking medications that affect the ANS were excluded. Each participant completed the composite autonomic symptoms score (COMPASS-31) [15] questionnaire and provided their clinical history. Information about COVID-19 vaccination was documented during the interview.

Blood pressure was measured three times using a standard sphygmomanometer after ten minutes in the supine position. Then, patients were asked to stand, and measurements were repeated three times. The average of the supine and standing readings was calculated.

### 2.2. Electrocardiographic Recording and Heart Rate Variability

All subjects wore a chest band (Bioharness 3.0, Zephyr Technology, Annapolis, MD, USA) for electrocardiogram recording in a quiet environment. Data were initially recorded while the subject was supine for 10 min, followed by 10 min during active standing. During data collection, the participants were instructed to stay still and relaxed, breathe naturally, and avoid speaking or falling asleep. All QRS complexes were identified to calculate heart rate variability over a 5 min segment at the end of each position using validated software (Kubios HRV version 2.0).

Time-domain indices, namely mean NN (average RR interval), SDNN (standard deviation of the RR interval), pNN50 (percentage of successive RR intervals with a difference greater than 50 ms), and RMSSD (root mean square of successive differences), were measured. The spectral power density was also obtained to estimate frequency-domain indices: mean power in the low-frequency band (LF, 0.4 to 0.15 Hz), associated with both the sympathetic and parasympathetic nervous systems, and mean power in the high-frequency band (HF, 0.15 to 0.4 Hz), linked to the parasympathetic system [16]. These indices were obtained in absolute units (ms^2^) and normalized units (nu) following the established guidelines [16]. Normalized units were obtained as follows: LF or HF normalized (nu) = [LF or HF (ms^2^)] × 100/(total power (ms^2^) − mean power in the very-low-frequency band (ms^2^) [16,17]. The LF/HF ratio, interpreted as an estimate of the balance between the sympathetic and parasympathetic nervous systems, was also calculated [18]. Although the interpretation of LF/HF has been widely debated [19,20], the increase in LF/HF in response to active standing is known to reflect the physiological response with increased sympathetic predominance due to a simultaneous decrease in parasympathetic modulation and increased sympathetic modulation towards the heart [16,21]. The estimated respiratory rate was also obtained from the frequency of the highest-power peak in the HF band. The power spectrum was calculated using Welch’s periodogram, an overlapping-windowed Fourier transform method.

For nonlinear analysis, geometric methods, such as the Poincaré plot, were used to obtain the HRV measurements [22]. These were performed by measuring the dispersions of RR intervals, which involved analyzing the HRV quantitatively by calculating the beat-to-beat standard deviation (SD). With this, SD1 reflects short-term changes in the RR interval driven by modulation of the parasympathetic nervous system on the sinoatrial node, and SD2 measures long-term changes in the RR interval, which are influenced by the parasympathetic and sympathetic systems [23]. Since SD1 measures the standard deviation of changes between consecutive heartbeats, it reflects the fastest beat-to-beat changes we could identify, which depend on the average heart rate. Conversely, SD2 exhibits small beat-to-beat changes that accumulate over long time scales, with slow, large-amplitude fluctuations increasing the standard deviation along the identity line. Therefore, SD2 is likely to reflect low- and very-low-frequency oscillations [23]. The relationship between the short-term and the long-term intervals was defined as SD1/SD2. This ratio indicates the proportion of fast (beat-to-beat) changes relative to slow HRV fluctuations, which is visually represented by the ellipse’s eccentricity formed by SD1 and SD2. Conditions with intense parasympathetic modulation, such as the supine position, result in large SD1 values, thereby widening the ellipse and increasing SD1/SD2 [23].

Additionally, the detrended fluctuation analysis method was used to calculate the scaling exponent, in which local trends are eliminated from the HRV fluctuations to estimate the average root mean square of the fluctuations for different time scales between 4 and 11 heartbeats. Then, the short-term scaling index (α_1_) is defined as the slope of a linear fitting of this average root mean square [24]. α_1_ quantifies the irregularity of the HRV behavior, from values close to 0.5 indicating highly irregular behavior to values larger than 1.0 associated with smoother (more regular) behavior. Physiological stimuli that produce sympathetic activation, such as active standing, increase the value of α_1_ [25]. Also, sample entropy was calculated according to Richman et al.’s method [26]. Sample entropy estimates the degree of regularity in the dynamic behavior of the RR intervals, with larger HRV and lower heart rate, and higher values of sample entropy are observed [27].

### 2.3. Biochemical Analysis

After 10 h of fasting, venous blood was drawn to obtain serum and plasma. Lipid profiles (total cholesterol, triglycerides, HDL-C, LDL-C), glucose levels, creatinine, uric acid, alanine aminotransferase, aspartate aminotransferase, gamma-glutamyl transpeptidase, and high-sensitivity C-reactive protein (Hs-CRP) were measured in plasma using automated photometry. The complete blood count was taken using fluorescent flow cytometry, photometry, and electrical impedance methods.

### 2.4. Statistical Analysis

Categorical variables were expressed as absolute values and percentages and compared between groups using a Chi-squared test. The Kolmogorov–Smirnov test assessed the normal distribution of quantitative variables. Variables with a normal distribution, whether in their original units or log-transformed, are shown as mean ± standard deviation and compared using Student’s *t*-test (for two groups) or ANOVA for repeated measures, with comparisons between groups (long COVID vs. healthy controls) and within groups (supine position vs. active standing). Post hoc comparisons were performed with the Bonferroni correction. All other quantitative variables are reported as median (interquartile range) and compared using the Mann–Whitney U test or the Wilcoxon test.

A *p*-value < 0.05 was considered significant. The analysis was performed using SPSS version 21.0.

## 3. Results

### 3.1. General Characteristics

Table 1 shows the clinical characteristics of 31 patients with long COVID and 29 healthy controls. Although most participants with long COVID were women, the difference between the groups was not statistically significant. The long COVID group had significantly more SARS-CoV-2 infections than the control group. As expected from the study design, a higher Composite Autonomic Symptoms Score-31 (COMPASS-31) was observed in the long COVID group compared with the control group. All other variables were similar between groups. The time elapsed from SARS-CoV-2 infection to testing in the long COVID group was 573 ± 289 days. All biochemical values were similar between the groups, ruling out comorbidities (Supplementary Material, Table S1).

**Figure 2:**
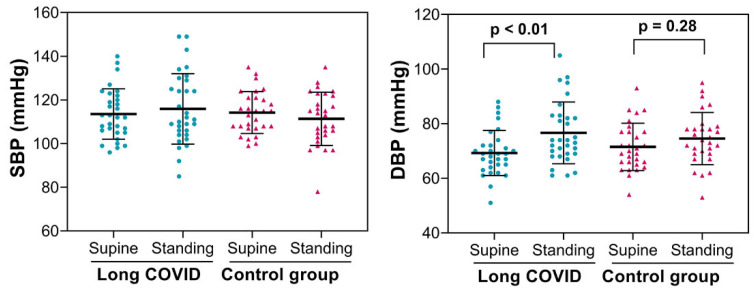
Systolic and diastolic blood pressure were measured in 31 patients with long COVID and 29 asymptomatic controls. *p* = *p*-value (ANOVA for repeated measures).

Most long COVID patients (85%) experienced orthostatic intolerance symptoms such as dizziness, pre-syncope, and syncope during their daily lives. Interestingly, none of them showed symptoms during active standing. The SBP results for both positions showed no significant differences between groups (long COVID versus control) or within groups (supine position vs. active standing), but DBP increased noticeably in long COVID patients during active standing compared to the control group (Figure 2). None of our patients had initial or delayed orthostatic hypotension or postural orthostatic tachycardia syndrome.

### 3.2. Heart Rate Variability Indices

**Figure 3:**
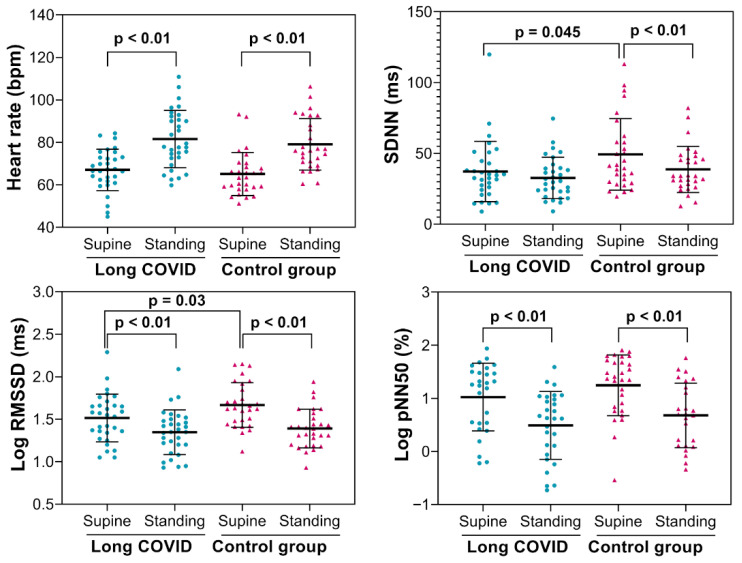
Heart rate and time-domain HRV indices were measured in 31 patients with long COVID and 29 asymptomatic controls. SDNN: standard deviation of the RR interval; pNN50: percentage of successive RR intervals with a difference greater than 50 ms; RMSSD: root mean square of successive differences. Log: logarithm of RMSSD or pNN50. *p*: *p*-value (ANOVA for repeated measures).

Heart rate and time domain HRV indices are displayed in Figure 3. Baseline (supine position) values of the standard deviation of normal-to-normal intervals (SDNN) and the root mean square of successive differences (RMSSD) were lower in long COVID patients compared to control participants.

**Figure 4:**
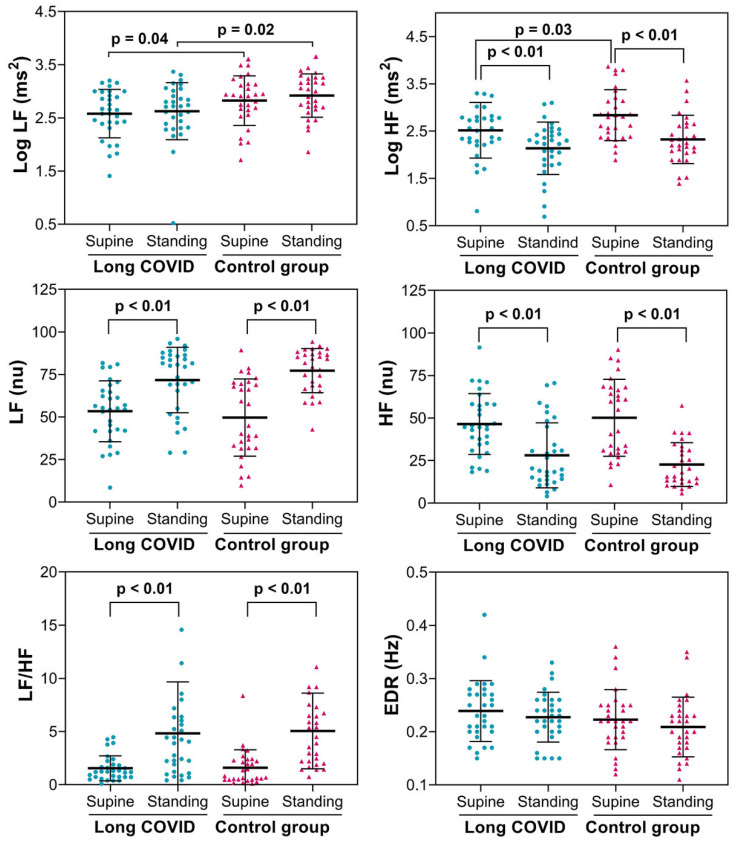
HRV spectral indices were measured in 31 patients with long COVID and 29 asymptomatic controls. HF: high frequency (power spectrum in the frequency range of 0.15–0.4); LF: low frequency (power spectrum in the frequency range of 0.04–0.15); LF/HF: ratio LF/HF; Log: logarithm from a ratio of LF to HF power. EDR: ECG-derived respiration. *p*: *p*-value (ANOVA for repeated measures).

By examining the HRV spectral analysis (Figure 4), we found that in a supine position, the mean power of low-frequency (LF) and high-frequency (HF) components in absolute values (ms^2^) was lower in long COVID patients compared to the controls. While standing, LF (ms^2^) remained lower in the long COVID group than in the control group. During active standing, both groups showed similar responses, marked by a decrease in HF (ms^2^ and nu). LF (nu) and the LF/HF ratio increased. The average respiratory rate, estimated from the power spectrum, showed no significant differences between the groups or in response to active standing.

**Figure 5:**
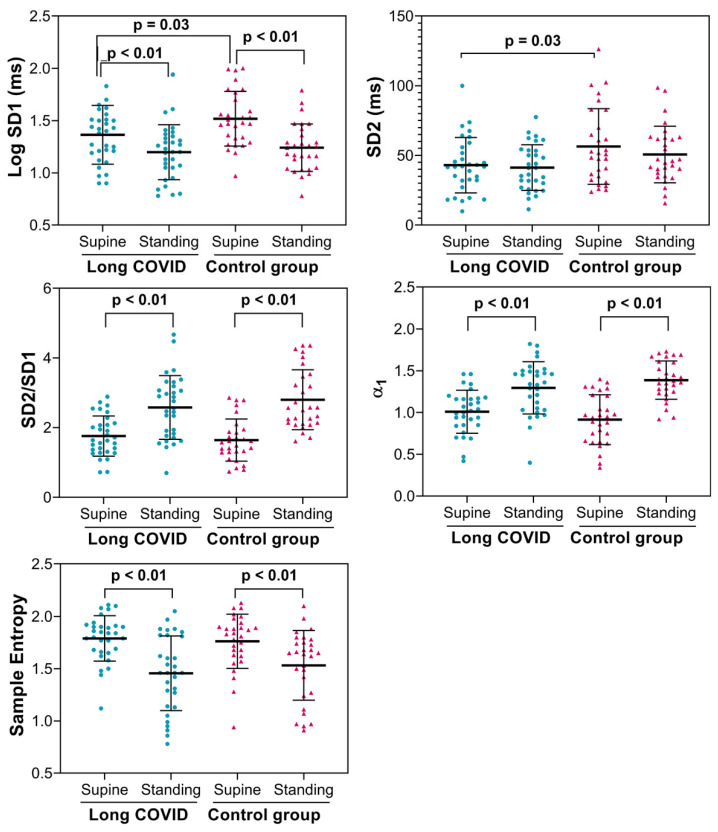
Nonlinear HRV indices were measured in 31 patients with long COVID and 29 asymptomatic controls. SD1: Poincaré plot standard deviation perpendicular to the line of identity; SD2: Poincaré plot standard deviation along the line of identity; SD1/SD2, ratio of SD1 to SD2; Alfa 1: slope of the linear interpolation of the spectrum in a log–log scale (f ≤ 0.01 Hz); Log: logarithm. *p*: *p*-value (ANOVA for repeated measures).

Regarding the nonlinear HRV indices shown in Figure 5, in the supine position, the short-term standard deviation (SD1) and the long-term standard deviation (SD2) were lower in patients with long COVID than in the controls. At the same time, SD2 remained lower in the long COVID group during active standing. In response to active standing, SD1 and sample entropy decreased, and the SD1/SD2 ratio and the short-term scaling index (α1) increased in both groups.

## 4. Discussion

Heart rate variability is a simple, non-invasive, and validated method for evaluating the autonomic nervous system. The evaluation of HRV using ECG can be performed using several approaches, including time- and frequency-domain analyses and nonlinear methods. Power spectral analysis of heart rate provides a quantitative measure of cardiac autonomic modulation. However, it has notable limitations in the low-frequency band, especially compared to parasympathetic activity at a high frequency, which is more specific [16]. HRV has been employed in patients with long COVID, and different responses have been observed.

This study examined HRV during an active orthostatic challenge in patients with long COVID who exhibit orthostatic intolerance, compared with asymptomatic control subjects. We found that SDNN, RMSSD, LF (ms^2^), HF (ms^2^), SD1, and SD2 at rest were significantly lower in the symptomatic long COVID patients than in the asymptomatic group, indicating reduced HRV. However, all other indices were similar between the groups. These findings differ partially from the results in other studies using long-term HRV. Karakayali et al. assessed autonomic function with HRV in patients who were symptomatic or asymptomatic after COVID-19. Symptomatic patients exhibited increased variability, demonstrated by higher pNN50, SD1, and SD2, compared to asymptomatic COVID-19 patients, while other HRV indices were similar between groups [28].

Asarcihli et al. analyzed autonomic function using HRV indices in patients with palpitations studied in the post-COVID period, also using 24 h Holter, and compared them with the healthy controls. Time-domain indices of HRV (SDNN and RMSSD) were significantly higher in post-COVID patients. Among frequency-domain indices, HF (ms^2^) was higher in long COVID patients [29]. These long-term evaluations, using 24 h ambulatory Holter recordings, encompass various conditions (e.g., awake and sleep periods, changes in body position) and uncontrolled physical activity that may influence HRV due to multiple regulatory mechanisms. In contrast, we performed our HRV analysis during the resting supine position, followed by active standing in a controlled protocol that reduced potential confounding factors that could influence the physiological response. These methodological differences could explain the discrepancies between these studies.

Using a similar method to ours (short-term HRV at rest, i.e., 5 min recordings), Marques et al. compared symptomatic patients with long COVID who were hospitalized, those not hospitalized, and a healthy control group [9]. Interestingly, they found reduced parasympathetic modulation in patients with long COVID. Moreover, they found increased sympathetic modulation in long COVID patients hospitalized during the acute infection or when symptoms lasted for less than 3 months. This higher sympathetic modulation was not present in long COVID patients with symptoms for more than 3 months. These findings suggest that autonomic dysregulation may improve over time. Our findings align with the behavior of the last group, as the average post-infection time was 47 months in our patients, which may explain why we did not observe reduced parasympathetic and increased sympathetic modulation in long COVID patients, contrary to our expectations.

In our study, the number of infections in the long COVID group was higher than in the control group. Studies have shown that the risk of developing long COVID increases with each subsequent SARS-CoV-2 infection. Individuals who have been infected multiple times are more likely to develop long COVID than those who have been infected only once [30].

Long COVID manifests in various ways depending on the tissues affected by SARS-CoV-2. If the virus impacts the hypothalamus, it could cause dysautonomia. The most common autonomic symptom after COVID-19 is orthostatic intolerance [28]. As noted in our study, long COVID patients often experience symptoms such as palpitations, dizziness, near-syncope, and syncope when standing. Nevertheless, contrary to our expectations, active orthostatic challenge did not trigger symptoms of orthostatic intolerance or cardiovascular autonomic abnormalities in any of them. Our findings differ from those reported by Hira et al. [7] but are similar to those observed by Adler et al. [8]. Although most participants in our study with long COVID were women, the difference between groups was not statistically significant. Since our research focused on patients with symptoms of orthostatic intolerance, the higher proportion of women in the long COVID group might be explained by the greater prevalence of orthostatic intolerance among women, which seems to be due to differences in hemodynamic control and autonomic function compared to men [29].

Few studies have examined autonomic cardiovascular responses to orthostatic challenges in patients with COVID-19, all using the head-up tilt test (HUTT). It is well known that passive HUTT induces graded changes in the sympathovagal balance. Stute et al. studied symptomatic young adults who tested positive for SARS-CoV-2 with microneurography. Resting muscle sympathetic nerve activity (MSNA) was higher in patients recovering from COVID-19 than in age-matched controls. Consistent with their observations at rest, MSNA was higher in COVID-19 patients during the orthostatic challenge than in the controls, although the orthostatic response was similar between groups. Therefore, while MSNA was elevated at rest and during the challenge, the response to passive orthostasis remained intact following mild SARS-CoV-2 infection [31]. In our current study, using HRV, both groups showed increased heart rate and decreased RMSSD and PNN50 in response to active standing, while SDNN only decreased in the control group. During the orthostatic challenge, power spectral analysis of HRV revealed similar reactions in both groups, with decreased HF (ms2 and nu) and increased LF (nu) and LF/HF ratio, which is consistent with a decrease in the cardiac parasympathetic modulation and an increase in the sympathetic modulation that results in increased sympathetic predominance [16,21]. Although the active standing test has limited clinical value for assessing orthostatic tolerance, it helps elucidate the physiological response to standing. Contrary to Salem et al. [32], who reported postural hypotension in 39.3% of post-COVID patients during the blood pressure response to the HUTT maneuver, we did not observe this with active orthostatic challenge. In fact, SBP and DBP showed no significant differences between groups, although DBP increased noticeably in long COVID patients during active standing compared to the control group. Our findings suggest that long COVID patients also maintain an intact physiological response to active orthostasis after a prolonged recovery period from infection.

Da Silva et al. [10] used the HUTT in patients with long COVID. HRV indices at rest showed lower RMSSD and HF power compared to control counterparts. Similarly to our study, HUTT elicited a tachycardic response in both groups, and, as expected, the time domain results demonstrated a reduction in RMSSD during the HUTT in both controls and patients with long COVID. Looking at the power spectral analysis of HRV, HF power was, as expected, reduced by HUTT in control subjects. However, in contrast to our findings, HUTT did not affect the HF power (ms^2^) of RR spectra in patients with long COVID, although when patients were reassessed after 6 months, HF power was reduced by HUTT, again indicating the influence of time on autonomic regulation in long COVID [9]. In their study, when the long COVID patients were initially evaluated, the power of LF (nu) did not increase in response to tilting. However, upon reassessment after 6 months, LF increased in response to the orthostatic challenge. Moreover, in our study, the nonlinear HRV indices SD1 and SD2 were lower in long COVID patients than in the controls, reflecting lower variability in the short and long term, respectively [33]. Marques et al. [9] reported a decreased SD1 at rest in long COVID patients studied < 3 months after infection. Our results show that long COVID patients with orthostatic intolerance have decreased SD1 and SD2 compared to the controls, even after a long time post-infection. In response to orthostatic challenge, both groups show similar responses in nonlinear HRV indices (decrease in SD1 and sample entropy, while SD2/SD1 and α_1_ increased [25]), all consistent with a physiological response of more regular dynamic behavior in the RR intervals [34].

In summary, our study shows that compared with the controls at rest, HRV in patients with long COVID evaluated more than 6 months after SARS-CoV-2 infection exhibited lower RMSSD, SDNN, LF (ms^2^), HF (ms^2^), SD1, and SD2 than the controls. All other HRV indices were similar between groups. These findings align with some previous observations from the literature, which indicate a reduced HRV at rest in patients with long COVID examined earlier after infection [35]. Although we could interpret the reduced RMSSD and HF (ms^2^) as indicative of impaired parasympathetic modulation to the heart at rest [36], the spectral indices, particularly LF (nu), HF (nu), and LF/HF, did not demonstrate clear autonomic nervous system dysfunction. Conversely, our findings may reflect a recovery of cardiac autonomic modulation after a long period since infection [9,35].

Contrary to our expectations, despite decreased HRV indicated by some time-domain indices in the supine position, long COVID patients with mild-to-moderate infection did not show impairment in cardiac autonomic control during active standing. Cairo B et al. studied the long-term impact of COVID-19 on cardiorespiratory control and the baroreflex during active standing. They observed a lack of response of the frequency-domain HRV indices to the orthostatic challenge, only in those with more severe COVID-19 infection. This finding supports the conclusion that dysautonomia associated with COVID-19 is less pronounced in patients with less severe disease [37].

The mechanism behind reduced HRV in patients with long COVID remains unclear. This may be due to inflammation caused by the virus or immune dysregulation after viral exposure [38]. In acute infectious diseases, sympathetic activation triggers the release of inflammatory cytokines, and the vagal anti-inflammatory reflex acts to counterbalance this response [39]. Analysis of HRV has proven useful for early detection of acute inflammatory responses, helping to predict outcomes and prognosis in hospitalized COVID-19 patients [40].

### Strengths and Limitations

Our study has several limitations. It was a single-center, cross-sectional study with a small sample size, and the results are limited to patients with mild-to-moderate COVID-19 who were studied long after SARS-CoV-2 infection. A significant limitation was that we did not obtain initial HRV data during the early stage of this disease to compare with. All patients were successively recruited when they sought attention.

The rate and depth of breathing influence HRV indices. Although patients’ breathing was not controlled in this study, a prior study found that long COVID patients have reduced HRV at rest and during controlled breathing [10]. While baroreflex sensitivity plays a key role in the response to orthostasis, we did not use non-invasive blood pressure monitoring to evaluate it in this study. Beat-to-beat blood pressure measurement is the ideal tool for identifying patients with hemodynamic cardiovascular autonomic abnormalities.

The control group consists of healthy, non-infected individuals and patients who had COVID-19 without long COVID symptoms. Since no significant differences were found between the groups, they were combined into a single asymptomatic group. Information about confirmed SARS-CoV-2 infection through molecular testing was limited and self-reported, which could be a potential limitation. Moreover, the study participants are a mix of vaccinated and unvaccinated individuals with zero to multiple COVID-19 infections. With limited information on how recent the other infections/vaccinations are, this is an important confounding factor.

Our study included only patients with mild-to-moderate COVID-19 severity who were recruited after a prolonged post-infection period. Since long COVID patients who are less than 3 months post-infection show more noticeable HRV changes [9], our findings in long COVID patients with orthostatic intolerance would likely have been more significant if studied earlier or in those with more severe infection.

Another significant limitation is that an active orthostatic challenge has low sensitivity in provoking symptoms of orthostatic intolerance. Additional studies using HUTT are needed to clarify the symptoms of orthostatic intolerance in patients with long COVID.

An important strength of this study is that the control groups were selected simultaneously (COVID-19 era) to prevent bias. The sample was representative of the population studied, and, to the best of our knowledge, this is the first study to use HRV analysis to assess autonomic cardiac modulation during active standing in patients with long COVID and orthostatic intolerance.

## 5. Conclusions

In conclusion, an active orthostatic test did not reveal autonomic dysregulation in these long COVID patients, suggesting that cardiac autonomic function may have recovered over time after SARS-CoV-2 infection, which remains to be confirmed in future studies. An alternative explanation is that only patients with more severe COVID-19 infections may exhibit autonomic nervous system dysfunction during the active orthostatic challenge. However, this study shows decreased HRV in patients with orthostatic intolerance after COVID-19 in the supine position compared with asymptomatic controls. This finding builds on previous observations that did not specifically focus on orthostatic intolerance.

## Figures and Tables

**Table 1:** Clinical characteristics of the study population.

Variable	Long COVID	Controls	*p* *
	(n-31)	(n-29)	
Age (years)	36.6 ± 11.4	38.28 ± 13.3	0.62
Female n (%)	24 (70.6%)	14 (48.3%)	0.06
Weight (kg)	70.3 ± 16.4	72.0 ± 10.5	0.64
Height (mt)	1.63 ± 0.08	1.65 ± 0.08	0.32
BMI (Kg/m^2^)	26.2 ± 5.03	26.2 ± 2.86	0.99
SARS-CoV-2 infections			<0.01
Zero	0 (0%)	15 (52%)	
One	14 (45%)	5 (17%)	
Two	13 (42%)	4 (14%)	
Three	4 (13%)	5 (17%)	
SARS-CoV-2 vaccines			0.14
Zero	4 (13%)	1 (4%)	
One	7 (23%)	2 (7%)	
Two	7 (23%)	6 (21%)	
Three	10 (33%)	11 (40%)	
Four	3 (10%)	8 (28%)	
COMPASS-31	44.76 ± 14.45	13.71 ± 12.25	<0.01

BMI: body mass index, COMPASS-31: composite autonomic symptoms score-31. * Student *t*-test, Chi-square. Data are expressed by means ± SD and median (IQR).

## Data Availability

The raw data supporting this article’s conclusions will be available upon request to the corresponding author, provided the pertinent legal requirements are met.

## Supplementary Materials

The following supporting information can be downloaded at: https://www.mdpi.com/article/10.3390/biology15010001/s1, Table S1: Biochemical variables.

## Abbreviations

The following abbreviations are used in this manuscript:

HRV	Heart rate variability
SDNN	Standard deviation of the RR interval
pNN50	Percentage of successive RR intervals with a difference greater than 50 ms
RMSSD	Root mean square of successive differences
SD1	Standard deviation perpendicular to the line of identity in the Poincaré plot
SD2	Standard deviation along the line of identity in the Poincaré plot
HF	Mean power in the high-frequency range of 0.15–0.4 Hz
LF	Mean power in the low-frequency range of 0.04–0.15 Hz
RR	R-R interval, the time between consecutive QRS complexes
MSNA	Muscle sympathetic nerve activity
ms^2^	squared milliseconds
