# Supplementary material for: Long COVID Patients with Orthostatic Intolerance Have Reduced Heart Rate Variability and Preserved Physiological Response to Active Standing
Source: Biology (Basel). 2025 Dec 19;15(1):1. doi: 10.3390/biology15010001 (PMC12784901; doi:10.3390/biology15010001)
Supplement: Supplementary file 1 [file biology-15-00001-s001.zip › biology-3913187-supplementary.pdf]

Supplementary Material

Table S1 Biochemical variables

Variable	Long COVID (n - 31)	Controls (n - 29)	p*
Glucose (mg/dL)	91.7 ± 10.57	90.37 ± 5.45	0.53
Uric acid (mg/dL)	5.18 ± 1.43	5.87 ± 1.17	0.07
Creatinine (mg/dL)	0.80 ± 0.12	0.88 ± 0.19	0.07
Proteins (mg/dL)	7.2 ± 0.4	7.2 ± 0.3	0.46
Cholesterol total (mg/dL)	181.5 ± 27.2	178.9 ± 46.2	0.79
HDL-C (mg/dL)	49.0 ± 13.2	47.2 ± 11.1	0.58
LDL-C (mg/dL)	117.0 ± 24.1	114.7 ± 38.5	0.78
Triglycerides (mg/dl)	140.2 ± 74.8	128.6 ± 80.9	0.56
ALT (U/L)	25.9 ± 9.9	28.9 ± 6.6	0.17
AST (U/L)	29.2 ± 18.0	33.0 ± 17.4	0.40
Hemoglobin (g/dL)	15.1 ± 1.5	15.6 ± 1.7	0.27
Hematocrit (%)	46.2 ± 4.2	46.7 ± 4.2	0.62
Leukocytes (10*^{3}/μL) #	6.8 ± 1.7	6.6 ± 1.3	0.65
Lymphocytes (10*3/μL) #	2.2 ± 0.5	2.3 ± 0.5	0.48
Neutrophils (10*3/μL) #	3.9 ± 1.5	3.6 ± 1.0	0.38
Platelets (10*3/μL) #	297.1 ± 53.0	276.3 ± 42.6	0.10
Hs-CRP (mg/dL)	1.16(0.64-3.02)	1.39(0.52-4.29)	0.18

ALT: Alanine aminotransferase, AST: aspartate aminotransferase, HDL-C: high-density cholesterol, LDL-C: very low-density cholesterol. Hs-CRP: high-sensitivity C-reactive protein.
